# Meckel’s Diverticulitis With Localized Perforation: A Case Report and Literature Review

**DOI:** 10.7759/cureus.78737

**Published:** 2025-02-08

**Authors:** Morgan A Hatlovic, Kasen Anzelc, Reema Tominna, Harsangeet K Chan-Gill, Kristen Conrad-Schnetz

**Affiliations:** 1 Surgery, Lake Erie College of Osteopathic Medicine, Bradenton, USA; 2 Surgery, South Pointe Hospital Cleveland Clinic Foundation, Cleveland, USA

**Keywords:** abdominal pain, congenital, meckel’s diverticulitis, meckel's diverticulum, perforated meckel's diverticulum

## Abstract

Meckel’s diverticulum is the most common congenital anomaly of the gastrointestinal (GI) tract. It presents most frequently in patients under two years of age and more rarely in later adulthood. Complications of Meckel’s diverticulum are even less common and can be difficult to distinguish from other more common abdominal pathologies due to nonspecific signs and symptoms. As a result, less common disease presentations, such as Meckel’s diverticulitis with perforation, can be difficult to diagnose preoperatively, especially in the adult population. We discuss a case of a patient who presented with symptoms of acute appendicitis, which was later revealed to be Meckel’s diverticulum complicated by Meckel’s diverticulitis with localized perforation.

## Introduction

Meckel’s diverticulum is the most common congenital anomaly of the gastrointestinal (GI) tract [1]. It is due to the persistence of the vitelline or omphalomesenteric duct, which usually obliterates by week 5-8 of gestation [1-3]. The literature reports that the prevalence of Meckel’s diverticula in the population is around 2% [2-4]. Meckel's diverticulum is known to exhibit symptoms in only a small percentage of patients, ranging from 2-6%. Moreover, complications associated with Meckel's diverticulum are observed more frequently in male patients [2-4]. Due to its low prevalence in the general population and asymptomatic nature in most cases, encountering Meckel’s diverticulum is uncommon [2,4].

Meckel’s diverticulum is hypothesized to be most commonly seen in patients under two years of age due to it being a congenital anomaly [3]. The most common clinical presentation of Meckel’s diverticulum is bleeding due to ectopic tissue in the diverticulum, commonly of gastric origin. Bleeding from the GI tract is the most common complication associated with Meckel’s diverticulum in children but is rare in adults. GI obstruction is the most common complication in the adult population [1,5]. With 40% of symptomatic cases reported to occur before the age of 10 years, this congenital anomaly is often misdiagnosed preoperatively in adults [1,4]. In adults, symptoms can range from nausea and vomiting to nonspecific abdominal pain, which is frequently clinically indistinguishable from appendicitis [6,7]. Therefore, preoperative diagnosis of Meckel's diverticulum is often difficult to distinguish from other self-limiting diseases or medical emergencies.

We present a case of a 79-year-old female with Meckel’s diverticulum mimicking acute appendicitis, further complicated by Meckel’s diverticulitis with localized perforation.

## Case presentation

The patient was a 79-year-old female with a history of breast cancer, status post right lumpectomy followed by radiation therapy, and melanoma of left shoulder (T1N0M0), status post wide excision with more than 1 cm margin and reconstruction with advancement flap, who presented to the emergency department with acute abdominal pain of one day’s duration. The pain was localized to the right lower quadrant, sharp in nature, and exacerbated by movement. The patient had no nausea, vomiting, fever, diaphoresis, hematochezia, or constipation. Vital signs were notable for tachycardia with a pulse of 104 bpm (normal range: 60-100 bpm), low-grade fever of 37.9°C (100.2°F), and blood pressure of 130/56 mmHg. Labs revealed a leukocytosis of 11,400 cells per mm³ (reference range: 4,400-9,700 cells/mm³) and an elevated lipase level of 770 U/L (reference range: 73-393 U/L).

**Figure 1:**
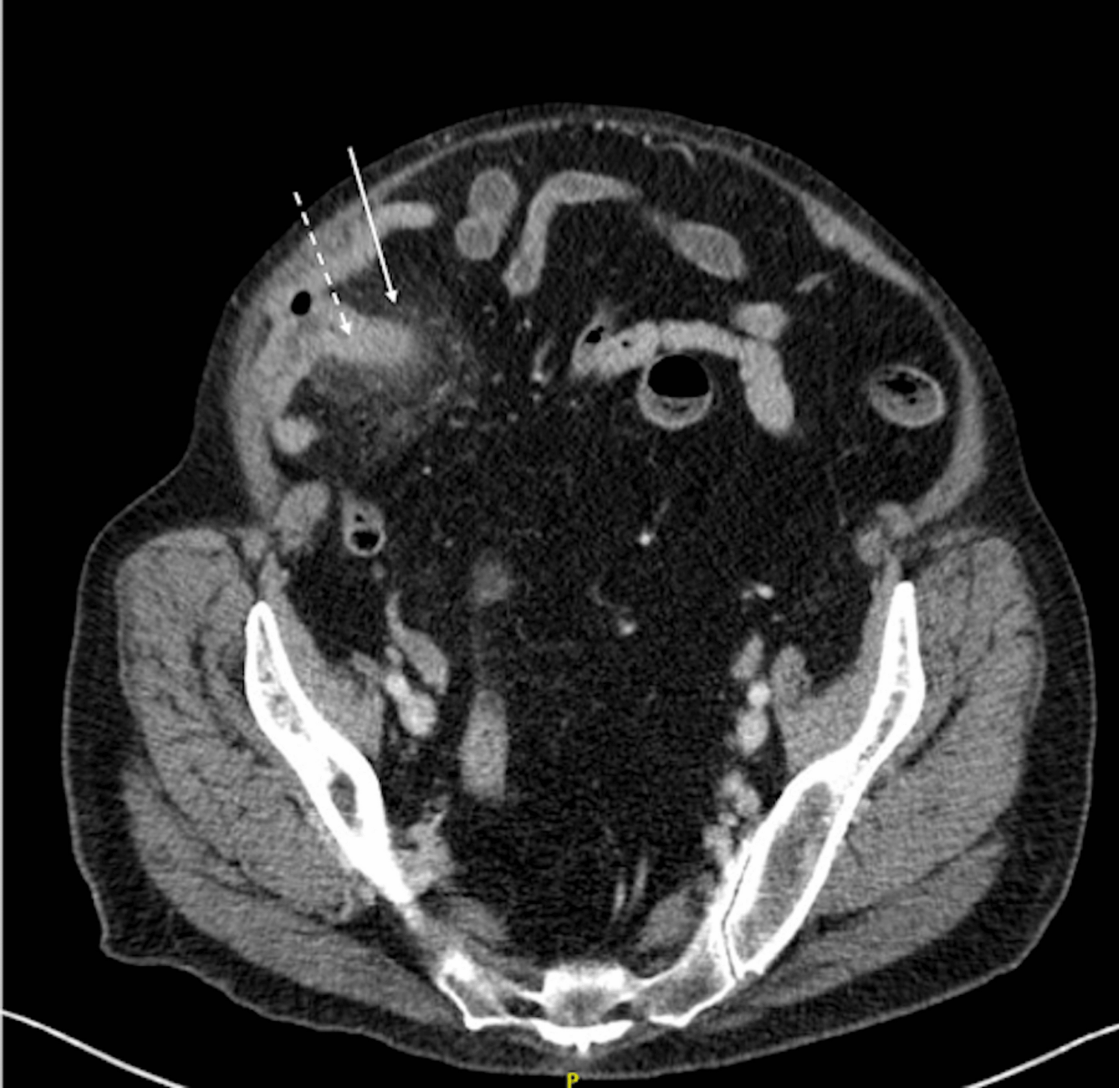
Axial plane of CT scan of abdomen and pelvis with IV contrast showing the appearance of an abnormally distended appendix (dotted white arrow) and abnormal fat stranding in adjacent soft tissues (white arrow). CT: Computed tomography; IV: Intravenous

**Figure 2:**
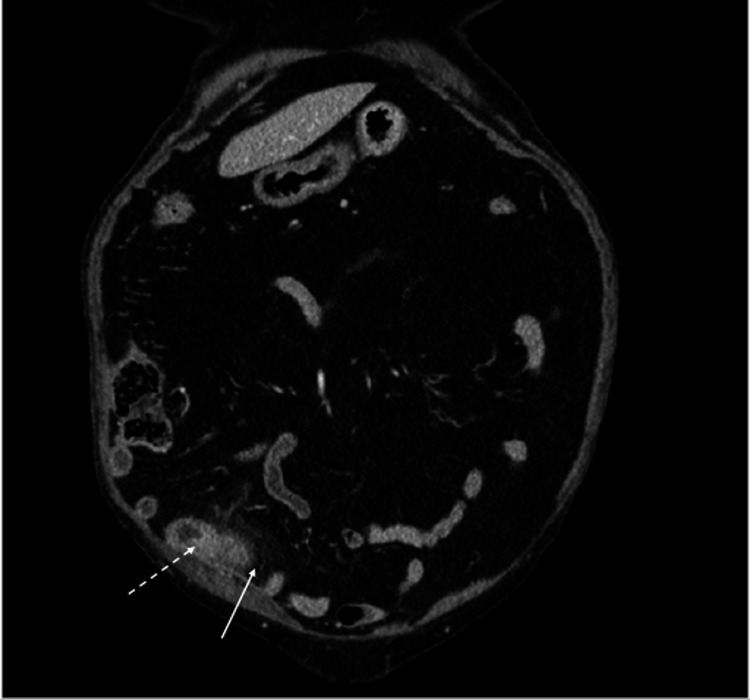
Coronal plane of CT scan of abdomen and pelvis with IV contrast showing the appearance of an abnormally distended appendix (dotted white arrow) and abnormal fat stranding in adjacent soft tissues (white arrow). CT: Computed tomography; IV: Intravenous

A computed tomography (CT) scan of the abdomen and pelvis with intravenous (IV) contrast showed what appeared to be an abnormally distended appendix measuring up to 13 mm in diameter, exhibiting abnormal fat stranding in adjacent soft tissues, with no signs of perforation, such as extraluminal gas or fluid collection (Figures 1-2). There was no evidence of bowel obstruction, and the gallbladder was surgically absent. The CT was deemed consistent with acute appendicitis.

The patient was taken to surgery for a laparoscopic appendectomy. Upon entering the abdomen, adhesions were revealed and subsequently bluntly taken down. The cecum was identified, and the appendix was seen and appeared normal. A segment of small bowel loops was adherent to the abdominal wall. Surgery was converted to an exploratory laparotomy and a midline incision was made. Further lysis of adhesions was performed using a combination of blunt and sharp dissection, which revealed a segment of an inflamed small bowel. After separating the bowel loops, a Meckel's diverticulum was identified as associated with phlegmon draining purulent fluid. A small 2 mm perforation in the associated segment of the small bowel was noted. The affected bowel was resected, measuring 15 x 3.5 cm. The mesentery was divided, an anastomosis was created, and the mesenteric defect was closed with a running vicryl stitch.

**Figure 3:**
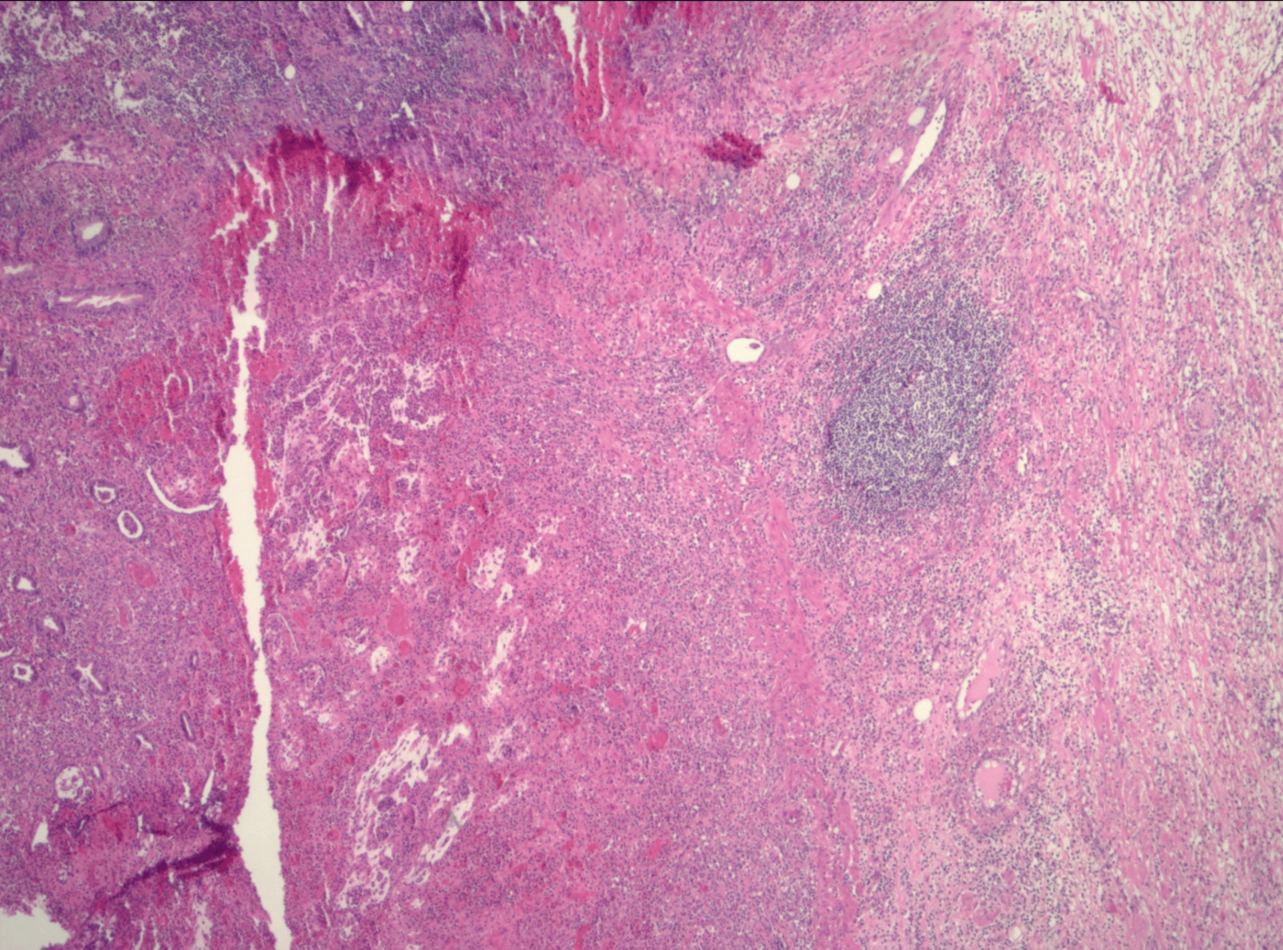
Pathology slide (H&E, x100) showing Meckel's diverticulum with extensive ulceration, acute inflammation, and residual glands. H&E: Hematoxylin and eosin

Surgical pathology was notable for the diverticulum measuring 3 x 3 x 2.5 cm, with a bowel wall thickness of 0.3 cm. Histology showed a Meckel's diverticulum with extensive ulceration, acute inflammation, and residual glands (Figure 3). The final pathology diagnosis was Meckel’s diverticulum with extensive ulceration and acute inflammation with associated acutely inflamed fibrovascular serosal adhesions.

The patient was seen by general surgery in the outpatient setting two weeks postoperatively. The patient was doing well and having bowel function with no complaints of abdominal pain or associated symptoms, including nausea or emesis. Staples from the midline incision were removed in the office.

## Discussion

Although Meckel’s diverticulum is known as the most common congenital anomaly of the gastrointestinal tract, its prevalence of approximately 2% in the general population makes it unlikely to be encountered in the clinical setting without accompanying symptoms [7,8]. Up to half of these diverticula exhibit ectopic mucosa, with gastric mucosa being the most common, followed by pancreatic mucosa [4]. Clinically, this ectopia can lead to ulceration and subsequent bleeding [7,9].

The most common complications of Meckel’s diverticulum range from bleeding in children to obstruction in adults. Other possibilities include intussusception, inflammation or diverticulitis, and perforation. Obstruction is more frequent among adults, comprising nearly 40% of symptomatic cases [3,7,9]. Diverticulitis, a clinical finding in about 13-53% of symptomatic cases, is more commonly seen in older patients and often misdiagnosed [9].

The complications of diverticulitis and perforation, as seen in our patient, occur at a rate of 20% together [3,5]. Perforation is thought to be a consequence of acute inflammation [2]. Perforation on imaging may show pneumoperitoneum, but diverticulitis and perforation combined are often indistinguishable from acute appendicitis until surgery due to overlapping symptoms, such as nausea and vomiting, right lower quadrant pain, and localized peritonitis [3,5]. If Meckel’s diverticulum is suspected, multiple tests may aid in the diagnosis, including plain radiography, barium studies, angiography, computed tomography, ultrasonography, and scintigraphy [9]. A CT scan is commonly used to aid in diagnosing Meckel's diverticulum in adults; however, due to its similar appearance to loops of small bowel on imaging, it is often not recognized as Meckel's diverticulum on imaging alone, as seen in our case. Technetium 99m scintigraphy can reveal ectopic gastric mucosa present in a diverticulum; however, in the absence of this ectopic tissue, it would not be helpful in the identification of Meckel's diverticulum [8]. Further, the examination of imaging may focus on observing the more commonly symptomatic structures, such as the appendix, rather than the less common Meckel’s diverticulum [8]. However, since the patient presentation of Meckel's diverticulum is often nonspecific and indistinguishable from other diagnoses, it may be overlooked [6].

A literature review revealed 11 cases of Meckel’s diverticulitis with perforation, which we have used for comparison with our patient. The signs and symptoms of this abdominal pathology included nausea with and without vomiting [4,10-15], strictly abdominal pain [16,17], and changes in bowel habits, such as the inability to pass stool or gas [18,19]. The associated pathology in these cases of combined Meckel’s diverticulitis and perforation included heterotopic mucosa [11,12,14,15,17,19], small bowel obstruction with torsion [10], fecalith or enterolith [13,16], and necrosis [18]. 

These cases show that the signs and symptoms of Meckel’s diverticulum with inflammation and perforation are not specific. As a result, establishing a preoperative diagnosis of Meckel’s diverticulum can be challenging. The preoperative diagnosis is often obscured by nonspecific signs and symptoms, including abdominal pain, nausea, vomiting, leukocytosis, and constipation, which indicate that an abdominal disease process is occurring but do not directly lead to the diagnosis of Meckel’s diverticulum pathology [7]. Meckel's diverticulum is frequently clinically indistinguishable from other intraabdominal conditions, such as appendicitis, inflammatory bowel disease, or causes of small bowel obstruction, due to nonspecific symptoms [6]. Consequently, fewer than 10% of complicated Meckel's diverticulum cases are diagnosed preoperatively; therefore, during surgery for appendicitis when a normal appendix is observed, the distal lumen should be thoroughly examined for the presence of a diverticulum [9]. 

Our patient presented with signs and symptoms of acute sharp right lower quadrant abdominal pain. Due to our patient lacking any other symptoms, diagnosing this condition was difficult preoperatively. On pathology, extensive ulceration was found in the inflamed Meckel’s diverticulum and no heterotopic mucosa was identified. This presentation was likely spontaneous or idiopathic due to the lack of heterotopic mucosa, fecalith or enterolith, obstruction, or necrosis. Although our patient had extensive ulceration and acute inflammation of the diverticulum, likely leading to localized perforation, the etiology of this overall presentation was unknown.

Surgical resection of symptomatic Meckel’s diverticulitis is the standard of care [5]. Options for resection include diverticulectomy or ileal resection [6]. Ileal resection should be considered in the presence of significant inflammation, tumor, intestinal ischemia, or perforation, as seen in our case [6,7]. With an inflamed ileum, end-to-end anastomosis is suggested [5]. Early postoperative complications include surgical site infection, prolonged ileus, and anastomotic leak with a cumulative incidence of 12% [6]. It remains challenging to determine the increased risk of complications with an incidentally found Meckel’s diverticulum intraoperatively. Although not recommended as the initial step in treatment, laparoscopy is an efficient way to localize this pathology for removal [5]. Our patient experienced an uneventful postoperative course without complications of surgery, making a full recovery back to their baseline of health.

## Conclusions

Complications of Meckel’s diverticula in later adulthood are rare. Numerous pathologies related to the small bowel or diverticulum may trigger Meckel’s diverticulitis with perforation. When considering the differential diagnosis of acute abdominal pain, investigation for Meckel’s diverticulum should be included. This approach may allow for expedited diagnosis and treatment, especially intraoperatively when findings are discordant with preoperative imaging and diagnosis.
